# Tests of HgCdTe Photodetectors Performances for Implementation on the MIST-A Instrument

**DOI:** 10.3390/s26072250

**Published:** 2026-04-05

**Authors:** Chiara Cencia, Eliana La Francesca, Mauro Ciarniello, Andrea Raponi, Fabrizio Capaccioni, Maria Cristina De Sanctis, Simone De Angelis, Michelangelo Formisano, Marco Ferrari, David Biondi, Angelo Boccaccini, Stefania Stefani, Giuseppe Piccioni, Alessandro Mura, Anna Galiano, Leonardo Tommasi, Clorinda Bartolo, Marcella Iuzzolino, Leda Bucciantini, Michele Dami, Giovanni Cossu, Stefano Nencioni, Angelo Olivieri, Eleonora Ammannito, Alessandra Tiberia, Gianrico Filacchione

**Affiliations:** 1 Institute for Space Astrophysics and Planetology (IAPS), National Institute of Astrophysics, 00133 Rome, Italy; eliana.lafrancesca@inaf.it (E.L.F.); mauro.ciarniello@inaf.it (M.C.); andrea.raponi@inaf.it (A.R.); fabrizio.capaccioni@inaf.it (F.C.); mariacristina.desanctis@inaf.it (M.C.D.S.); simone.deangelis@inaf.it (S.D.A.); michelangelo.formisano@inaf.it (M.F.); marco.ferrari@inaf.it (M.F.); david.biondi@inaf.it (D.B.); angelo.boccaccini@inaf.it (A.B.); stefania.stefani@inaf.it (S.S.); giuseppe.piccioni@iaps.inaf.it (G.P.); alessandro.mura@inaf.it (A.M.); anna.galiano@inaf.it (A.G.); 2 Department of Physics, Tor Vergata University, 00133 Rome, Italy; 3 Leonardo Company, 50013 Campi Bisenzio, Italy; leonardo.tommasi@leonardo.com (L.T.); clorinda.bartolo@leonardo.com (C.B.); marcella.iuzzolino.ext@leonardo.com (M.I.); leda.bucciantini@leonardo.com (L.B.); michele.dami@leonardo.com (M.D.); giovanniantonio.cossu.ext@leonardo.com (G.C.); stefano.nencioni@leonardo.com (S.N.); 4 Italian Space Agency, 00133 Rome, Italy; angelo.olivieri@asi.it (A.O.); eleonora.ammannito@asi.it (E.A.); alessandra.tiberia@asi.it (A.T.)

**Keywords:** HgCdTe, infrared (IR) sensors, photodiodes, sensor calibration, sensor testingand evaluation

## Abstract

The Middle-Wave Infrared Imaging Spectrometer for Target Asteroids (MIST-A) will be launched in 2028 aboard the Emirates Mission to the Asteroid belt (EMA) and will operate in the 2–5 μm spectral range to study the asteroids’ surface composition and thermo-physical properties. MIST-A’s Optical Head (OH) design is inherited from the Jovian IR Auroral Mapper (JIRAM), from which the instrument also received two spare Hybrid-Thinned Mercury-Cadmium-Telluride (MCT) photodetectors: the Engineering Model EM2 and the Flight Spare FS1. These are tested to assess their performance after a long period of storage. The laboratory setup for testing both detectors consists of a blackbody and a cryostat which houses the focal plane, maintained at temperatures of 85 K, its nominal operative temperature, and 90 K. Two sets of measurements are performed: (1) characterization of the dark current at different integration times (0 ms, 224 ms, 448 ms, 672 ms, 869 ms, 1120 ms); (2) verification of the detectors’ response linearity, measuring a blackbody at different temperatures (from 50 °C to 100 °C), including ambient temperature (25 °C, with the blackbody turned off). The results of these tests confirm that both models are fully operational and allow us to evaluate the consequences of the years of inactivity on their performance. Through a detailed analysis of the detectors’ properties and a comparison study with the results of the sensors’ first characterization performed by their producer in 2009, we come to the conclusion that both instruments are able to fulfill MIST-A’s scientific requirements. The FS1 displays a better performance with respect to the EM2 and for this has been selected as MIST-A’s Flight Model.

## 1. Introduction

MIST-A is the Middle-Wave Infrared Imaging Spectrometer for Target-Asteroids that will be launched aboard the MBR Explorer spacecraft as part of the Emirates Mission to the Asteroid Belt (EMA) [1]. The EMA Mission is currently undergoing a 6-year development phase and is planned to launch in March 2028. During a 7-year-long cruise towards the Main Belt—achieved through a series of gravitational assists around Venus, Mars and the Earth and solar electric propulsion arcs—the spacecraft will perform six fly-bys of as many primitive asteroids before reaching the main target of the mission, asteroid 269 Justitia, in 2035 and deploying a lander on its surface [2]. Justitia represents a target of particular interest due to the peculiar redness of its spectrum, which may be a clue to the presence of complex organic materials on its surface [3]. Through its planned observations, EMA aims to study the origin and dynamical evolution of Main Belt asteroids and to research the possibility of employing asteroids’ materials as resources for future space missions. In order to reach these goals, the MBR Explorer scientific payload will include a high-resolution camera, a thermal infrared camera, a thermal infrared spectrometer, and the MIST-A spectrometer [4]. The MIST-A payload was selected by the United Arab Emirates Space Agency (UAESA) back in 2023 through a Request for Information competitive call. The terms of collaboration between UAESA and the Italian Space Agency (ASI) are ruled by a cooperative agreement among the parties.

In this manuscript, we are going to describe the activities performed to evaluate the performances of the infrared (IR) detectors inherited from the previous JIRAM program after a 15-year-long period of storage. In Section 1 we give a description of the MIST-A instrument and in particular its optical design. Section 2 focuses on the laboratory setup and the measurements performed to check the IR detectors’ functionalities and performances. The results of our tests are presented in Section 3; the assessment of the dark current, order sorting filters junction dead zone, defective pixels, and response linearity is discussed in Section 4. The conclusions about these activities are reported in Section 5.

### 1.1. The MIST-A Instrument

MIST-A’s scientific objectives focus on the identification of the asteroids’ surface composition and thermophysical properties [5]. MIST-A operates in the 2–5 μm spectral range, where it can identify and map various classes of compounds relevant for primitive targets and additionally measure the target asteroids’ surface thermal emission. By repeating observations at varying illumination and viewing geometries during flybys and orbits at Justitia, spectrophotometric models applied to MIST-A data can be employed to derive the surface’s regolith composition and physical properties [6]. During the nominal science orbit at Justitia, MIST-A will observe the surface with a spatial resolution of about 16 m/px while during the flybys, the maximum spatial resolution at closest approach will be between 80 and 180 m/px.

### 1.2. MIST-A Design

MIST-A is composed of two units: the OH and the Electronics Unit (EU). The instrument inherits its optical layout from JIRAM [7], the spectrometer aboard NASA’s JUNO mission, although some changes to its original thermo-mechanical design have been introduced:

- While JIRAM operated on a spinning spacecraft, MIST-A is mounted on a steerable 3-axis platform, which required a conversion of the scan mirror’s actuator system from a de-spinning application to a scanning/pointing mechanism.
- The instrument’s electronics have been updated, particularly the readout electronics that have been optimized for fly-by operations, going from a rate of 2 acquisitions per minute on JIRAM to about 72 acquisitions per minute.
- The mechanical design has been adjusted to sustain the launchers’ vibration levels, and the thermal design has been made suitable for operating in the Main Belt environment and at the MBR Spacecraft temperatures. This included the replacement of one of JIRAM’s two passive radiators dedicated to cooling down the IR detector with an active cryocooler, which allows better thermal control.
- Finally, JIRAM’s Imaging Channel, designed for the observation of Jupiter’s auroral emissions and atmospheric thermal emission, has been removed as not necessary to fulfill MIST-A’s objectives. This grants a simplification of the mechanical design and an improvement of the spectrometer’s performance.

**Figure 1:**
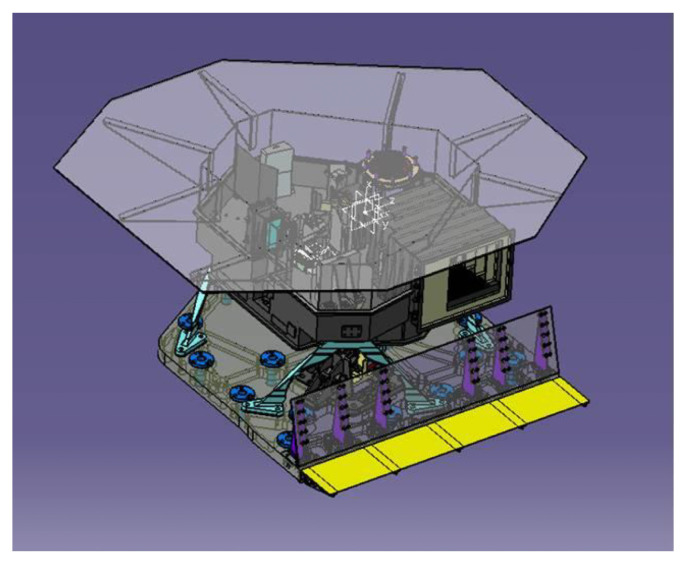
Layout of MIST-A optical head (Leonardo courtesy).

MIST-A’s OH (Figure 1) accommodates a modified Schmidt telescope (focal length of 160 mm, f/# = 3.7, aperture of 44 mm) connected to the entrance slit of a Littrow spectrometer where a flat grating (30.3 grooves/mm, blaze angle $\theta_{B}$ = 2.56°) is used as the dispersive element. The telescope is equipped at its entrance with a flat mirror on a 1-axis steerable mechanism for pointing and scanning. The mirror is also used to monitor the instrument’s response stability in flight by pointing the Internal Calibration Unit placed in proximity of the telescope’s entrance baffle.

**Figure 2:**
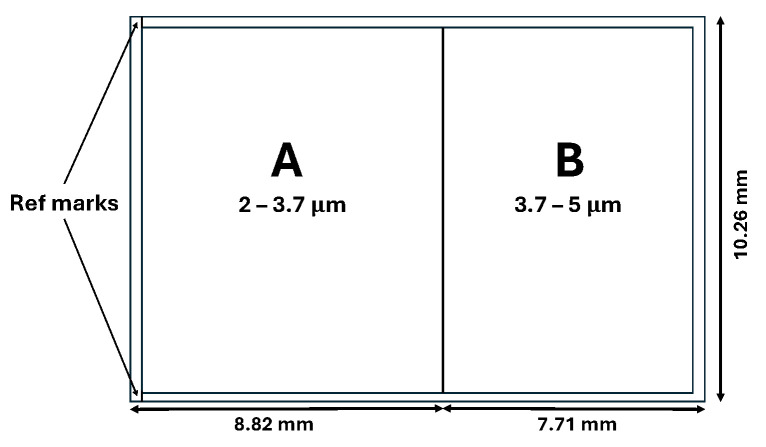
Layout of the order-sorting filters A and B placed on the IR detector. The spectral axis (bands) is along the horizontal axis, and the spatial axis (sample) along the vertical.

Other than JIRAM’s layout, MIST-A additionally relies on some of its spare elements. In particular, the spectrometer is equipped with one of JIRAM’s spare Hybrid-Thinned MCT photodetectors, manufactured by RVS Raytheon Video Systems, Goletha, CA, USA. The photosensitive array (270 × 438 pixels format, 40 μm pixel pitch, 2 Me^−^ full-well capacity) is mounted above a Complementary Metal-Oxide-Semiconductor (CMOS) readout integrated circuit (ROIC) and housed in a thermomechanical structure that also accommodates a cold shield, a light trap, and two order-sorting filters. Filters A (bandpass 2 μm ≤λ≤ 4 μm) and B (bandpass 3.5 μm ≤λ≤ 5.2 μm) are joined at the samples corresponding to about 3.7–3.8 µm on the spectral dispersion axis (Figure 2). Those filters are necessary to suppress the grating’s higher orders and thermal background with the aim of preserving spectral purity in the collected signal.

During the acquisition of science observations, the focal plane array (FPA) will be maintained at a cryogenic temperature of 85 K through an active cryocooler, while the mechanical structure will operate at temperatures ≤ 135 K and will be cooled by means of a passive radiator.

The instrument’s EU is responsible for performing tasks like managing the interface of the instrument to the spacecraft, acquiring and processing the raw data from the FPA, and controlling all of MIST-A’s functions. This is achieved through four boards the EU houses: the Power Converter and Distribution Unit; the Central Processing Unit Board, containing the Command and Process Control Unit and the Data Compression Unit; the Proximity Electronics, which is in charge to drive and digitize the FPA signal, and the auxiliary board necessary to command the scan mirror position and the temperature set point of the cryocooler.

Two models of JIRAM’s detector, the Engineering Model EM2 (EM hereinafter) and Flight Spare Model FS1 (FS hereinafter), are available. The sensors were first switched on at ambient temperatures, and after verifying that they were still operational, they were tested at cryogenic temperatures at the Leonardo laboratories of Campi Bisenzio (FI). The main aim of the tests is to assess the detectors’ performances after a long period of inactivity, particularly to check their responsivity, the number of defective pixels, and dark current with respect to the integration times and the detector’s temperature. The tests allow us to choose which detector to mount in the MIST-A instrument between the available models.

## 2. Materials and Methods

### 2.1. Laboratory Setup

**Figure 3:**
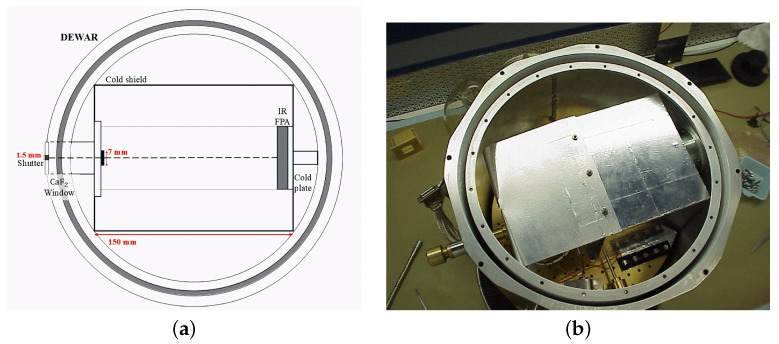
Outline (**a**) and picture (**b**) of the cryostat used during the tests on JIRAM EM and FS photodetectors.

The laboratory set-up used during the detectors’ tests is based on the same configuration employed for the characterization of the Visible InfraRed and Thermal Imaging Spectrometer (VIRTIS-M) [8] and JIRAM IR instruments. It consists of an optical bench which houses a liquid nitrogen (LN2)-cooled two-stage cryostat containing the detector, an extended blackbody (Santa Barbara Infrared (SBIR), Inc., Santa Barbara, CA, USA), and custom electronics, including the JIRAM Electrical Ground Support Equipment (EGSE) and the cryostat’s thermal controller (Figure 3). The FPAs are connected to the JIRAM Proximity Electronics Module (PEM), which allows powering the detector under test, sending telecommands, receiving and digitizing data and telemetry, and they are monitored and operated through its EGSE.

The focal plane under test is attached to a cold plate inside the cryostat, which maintains the detector at a stable temperature selectable between 80 K and 100 K thanks to a PID (Proportional-Integral-Derivative) controller. The internal configuration of the dewar was adjusted in order to reduce the amount of thermal background radiation incident on the FPA and increase the signal-to-noise ratio. This was achieved by setting the f-number of the dewar to 100 and adding a cold shield, provided with a 7 mm aperture, around the detector. The focal plane is located in front of the cold stop aperture, at a distance of 150 mm. The aperture has a diameter of 1.50 mm and is equipped with a filter wheel. The wheel is thermalized to the same temperature as the cold shield, and it is settable in five different configurations; the closed aperture configuration is to be used for dark current measurements. The internal configuration of the cryostat is shown in Figure 3.

During each acquisition, the temperature of the focal plane is measured directly by the EGSE thanks to a temperature sensor (Lakeshore diode) mounted onto the detector. In order to continuously monitor the temperature of the set-up, three PT100 temperature sensors were opportunely placed on the cold shield, the filter wheel, and the cold plate. The latter was also used as a reference point for the active PID controller employed to stabilize the temperature of the setup. A PT100 sensor was additionally mounted on the internal wall of the dewar to measure the lowest temperature achieved during the tests (80 K) and to monitor the level of LN2 in the dewar’s internal chamber.

### 2.2. Data Acquisition

While the detector’s full format is 270 samples × 438 bands, the MIST-A optical design uses only a subset of 256 samples × 336 bands. Accordingly, the EGSE is designed to acquire such a format by selecting an active window. For this reason, to reconstruct a complete frame of the detector, necessary to assess the overall detector’s response, the measurements are split into four windows of 336 × 256 pixels to be observed in following sequence:

- Window 0,0: range of 0:335 bands and 0:255 samples;
- Window 0,14: range of 0:335 bands and 14:269 samples;
- Window 102,0: range of 102:437 bands and 0:255 samples;
- Window 102,14: range of 102:437 bands and 14:269 samples.

The EGSE takes 30 s for a single frame acquisition and registration; 3 frames are recorded for each window, resulting in an acquisition time of 1 min and 30 s. The 256 × 336 subframes are then opportunely rearranged, averaging the overlapping regions, to recreate the detectors’ full area (270 × 438).

The test measurements are carried out for a detector temperature of 85 K and then repeated at 90 K. The latter analysis is performed in order to study any variation in a warmer case and to compare the results of our evaluation with those obtained during the compliance tests of the focal planes in 2009, when they were tested at 90 K by their manufacturer.

The signal values obtained in DN (ADU, *Analog Digital Units*) can be converted into volts using the instrument conversion Formula (1).

(1) $$V\left(in_{spe}\right)=\frac{\frac{ADU\left[2\left(speREF_{p}-speREF_{m}\right)/16384\right]-Offset\left(adc_{spe}\right)}{G(adc_{spe})}-Offset_{x}\left(ampl_{spe}\right)}{G_{x}\left(ampl_{spe}\right)}$$

Our measurements are performed in a low-gain setup configuration, so we consider the following parameter values set for the analog-to-digital (ADC) converter:

- *speREF_p_* = 1.2486 V;
- *speREF_m_* = 0.0095 mV;
- *Offset(adc_spe_)* = 1.844 mV;
- *G(adc_spe_)* = 0.993;
- *OffsetLow(ampl)* = 105.62 mV (728.99 DN) @ $V_{in}$ = 3.20081 V;
- *GLow(ampl_spe_)* = 0.76798.

where *speREF_p_* and *speREF_m_* are the voltage reference values of the detector; *Offset(adc_spe_)* and *G(adc_spe_)* represent the Offset and Gain of the ADC converter; *OffsetLow(ampl)* and *GLow(ampl_spe_)* are the Offset and Gain introduced by the amplifier working in Low Gain Mode. The factor 16,384 comes from the 14-bit ADC conversion. The Volt values can then be converted into units of photoelectrons using the nominal transimpedance value of Z = $1.15\times 10^{-6}\;{V/e}^{-}$.

## 3. Results

The results of the tests are compared to the performances of the detectors when they were first examined in 2009 by their manufacturer in order to evaluate any possible deterioration.

### 3.1. Dark Current Measurements

During the DC measurements, the filter wheel is placed in the closed window configuration. The measurements are performed at different integration times, starting at approximately 0 s (we consider the shortest exposure time achievable by the PEM, which is of a few microseconds, to be null) and then progressively increasing: 224 ms, 448 ms, 672 ms, 869 ms, and 1120 ms. These measures include, in addition to the dark current signal, also the thermal background generated by the cold shield and the closed shutter. However, considering both of these components are thermalized at a temperature of 77 K, which remains constant through all cases—for both the EM and the FS tests, at both detector temperatures of 85 K and 90 K—their contribution can be overlooked when studying the difference in the DC trends.

**Figure 4:**
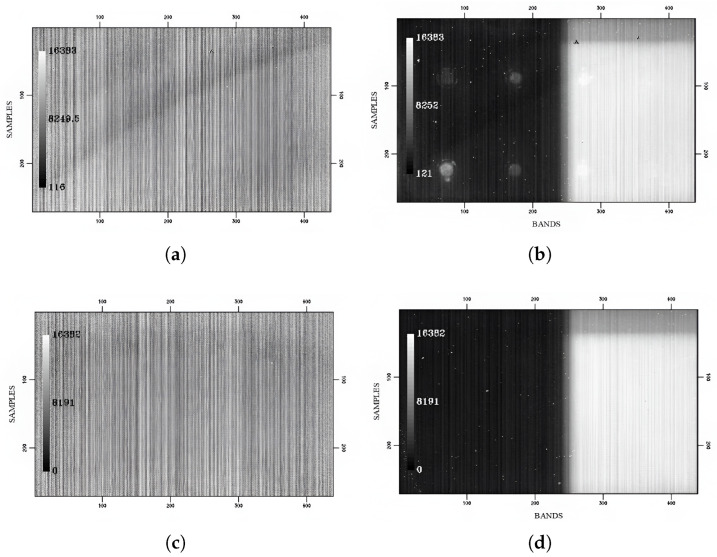
(**a**) Dark current measured for the EM at T = 85 K with an exposure time t = 0 ms. (**b**) Same as (**a**) but for t = 1120 ms. (**c**) Dark current measured for the FS at T = 85 K with an exposure time t = 0 ms. (**d**) Same as (**c**) but for t = 1120 ms.

The DC images acquired by the EM show a series of arc structures across the entire focal plane, which are particularly visible on the measurements at minimum integration time, where the fixed-pattern noise is predominant. Increasing the integration time, the effect of the two filters starts becoming noticeable, with the B filter (on the right side of the FPA) collecting a significantly more intense signal than the A filter. At longer times, we can also observe eight circular patterns characterized by higher (with respect to the local average) DC values (Figure 4a,b). The DC images acquired by the FS only show a decrease of the signal (step function along the sample direction at all bands) on the top of the image, particularly visible in the B filter region, which is also present in the EM images (Figure 4c,d).

**Figure 5:**
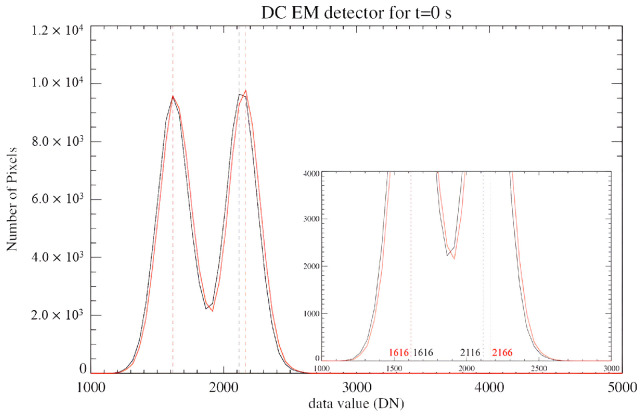
Histogram representing the DC values (in DN) recorded on the pixels across the focal plane for t*_int_* = 0 ms by the EM detector at 85 K (black curve) and 90 K (red curve). The zoomed-up sub-panel shows the most frequent DC values recorded on the odd and even bands for each case.

**Figure 6:**
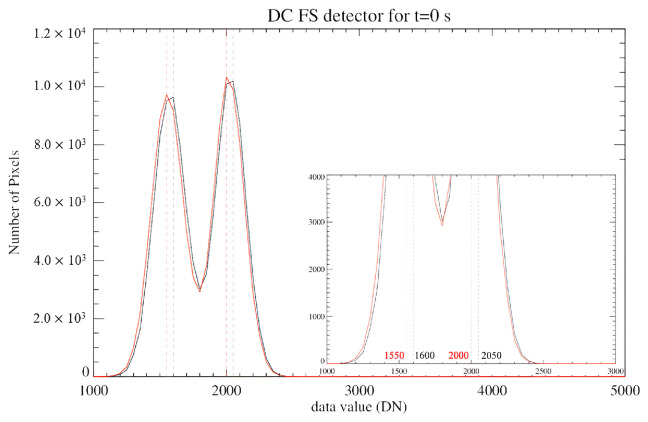
Same as Figure 5 but for the FS detector.

The dark current measurements were also used to detect the presence of an odd-even effect affecting the focal planes. The odd-even effect is a high-frequency noise introduced by the detector’s multiplexer [9], that manifests itself as a persistent offset between the digital counts registered on the even and odd bands on the detector. This effect is more visible when the signal is low (see vertical stripes in Figure 4 top panels). In order to check for the presence and evolution of this effect, DC histograms are compiled for both EM and FS detectors. The presence of an odd-even effect can be clearly inferred from the histograms of the DC signal recorded at the shortest integration time (Figure 5 and Figure 6).

The two peaks on the distributions represent the current measured on the two sets of bands; the DC values corresponding to the peaks of each distribution are reported in Table 1.

Therefore, we measure that the even bands record on average 22–25% digital counts less than the odd bands.

**Figure 7:**
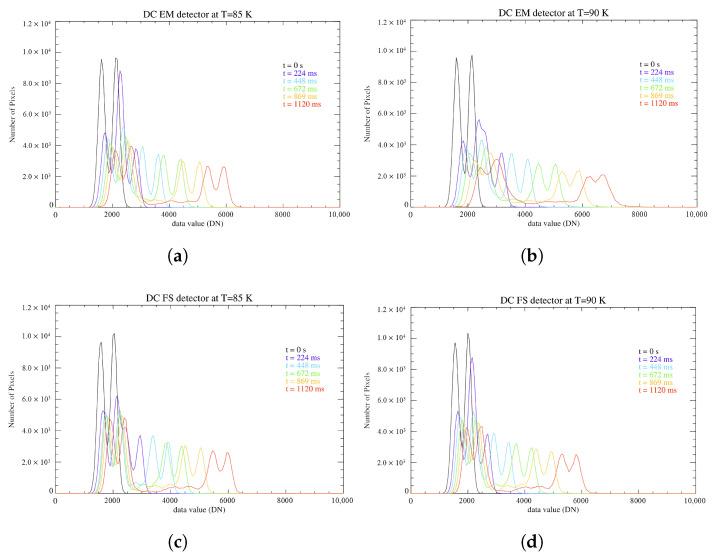
(**a**) Histograms representing the DC values (in DN) recorded on the pixels across the focal plane for different integration times by the EM detector at 85 K. (**b**) Same but for the detector at 90 K. (**c**) Histograms representing the DC values recorded on the pixels across the focal plane for different integration times by the FS detector at 85 K. (**d**) Same but for the detector at 90 K.

Compiling the histograms for the rest of the sets of measurements (Figure 7), we can observe the difference in the recorded DC on the A and B filters, with the latter registering a more intense signal, depicted by two distinct large peaks which grow further apart as the divergence increases with the integration time. The histogram at 224 ms exhibits three main peaks instead, due to the overlapping of the signal collected on the odd bands of the A filter and the even bands on the B filter, represented by the middle peak.

**Figure 8:**
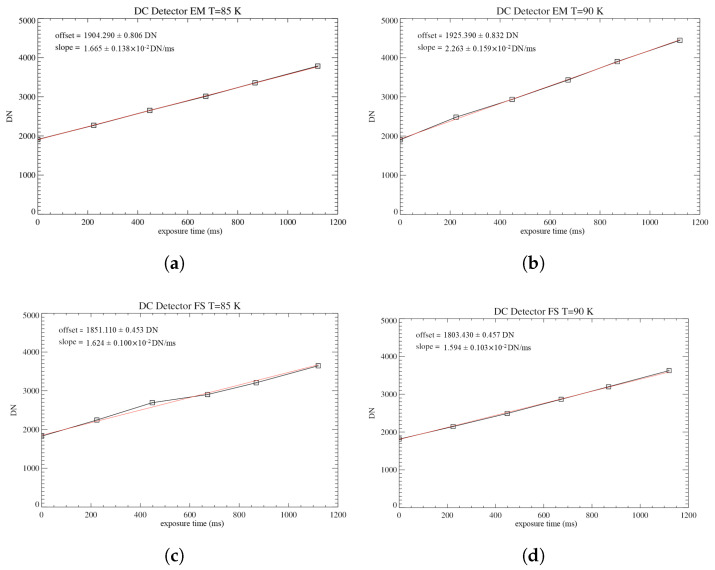
Average DC measured by the EM at T = 85 (**a**), 90 K (**b**), plotted as a function of the exposure times. The red curve shows the linear fit applied. The error bars represent the standard deviation of the mean, but due to it being in the order of 1 DN, they are not visible. (**c**,**d**) Same as (**a**,**b**) but for the FS model.

In order to study and compare the DC effect on the detectors, the collected data are averaged. To take into consideration both the odd-even effect and the difference in signal intensity on the A and B filters, this analysis was performed separately for the four populations of pixels representing the odd and even bands on each of the filters. A weighted average of the four mean values is compiled, and the results are reported on a DC vs. integration time plot. In all cases, the DC shows a smooth linear trend, in accordance with the results of the odd-even effect analysis; a linear fit is applied to measure the curve’s average slope and offset (Figure 8).

The results of the fits (Table 2), performed at T = 85 and 90 K, show a steeper growth of the DC signal for the EM detector, which will present higher noise, consequently affecting its performance more significantly with respect to the FS model.

### 3.2. Order Sorting Filters Dead Zone

As mentioned above, JIRAM’s detectors are equipped with two order sorting filters, A and B, necessary to suppress the light reflected by the grating on high orders. The filters collectively cover the 2–5 μm operative spectral range of MIST-A and are joined at about 3.8 μm on the spectral axis. The region of the focal plane affected by the junction between the two filters shows a strong decline in the response of the pixels.

**Figure 9:**
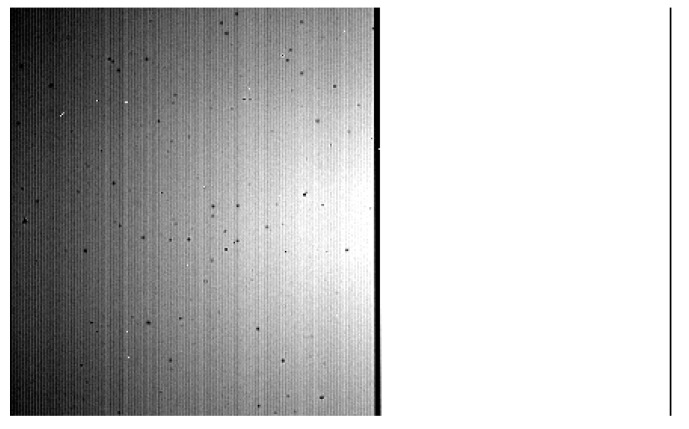
Black body signal at T = 100 °C recorded by the FS detector at T = 90 K. The dark vertical region delineates the dead zone between the two filters. The B filter area (in white, on the right) is saturated.

To define the precise interval of bands delineating this ‘dead zone’, we analyze the signal measured on the pixels when observing the black body at T = 100 °C. This configuration in particular is preferred for this study because at this temperature the B filter has entirely saturated and the gap between the two filters is clearly visible (Figure 9).

**Figure 10:**
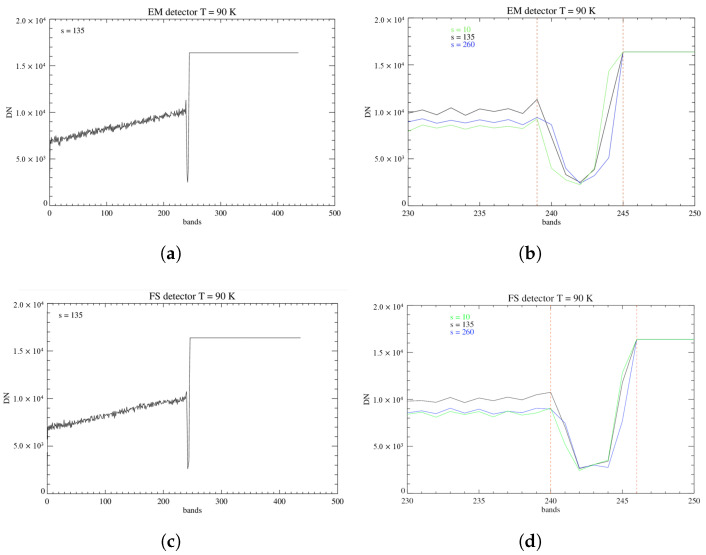
(**a**) Response of the EM focal plane, at T = 90 K, across the boresight (s = 135) and along the bands of the FPA observing the black body at T = 100 °C. (**b**) Response of the EM focal plane on samples 10, 135, 260. The dashed red lines mark the edges of the dead zone. (**c**,**d**) Same as (**a**,**b**) but for the FS detector at T = 90 K.

We observe the response of the pixels across 3 samples on the focal plane: s = 10, 135 (corresponding to the boresight), and 260. The different heights are chosen in order to characterize the junction across the entire detector (Figure 10).

From the measured response, we infer that the dead zone covers a range of 7 bands on both detectors. More precisely, it interests the bands intervals of 239–245 on the EM and 240–246 on the FS. Pixels belonging to these columns were excluded from successive analyses.

### 3.3. Defective Pixels

A first look at the DC frames reveals the presence of a notable amount of defective pixels across both focal planes. The detection of these defects is based on the comparison of the DC signal measured on the single pixels to the average DC registered by the detector. In order to have a more accurate analysis, the A-B filter regions are considered individually, due to their significant difference in the DN values of the DC registered. The odd-even effect is also taken into consideration, conducting the study of the odd and even bands on the focal plane separately. The number of defective pixels is thus estimated independently and finally summed. During the procedure, the DC measured across each filter region on the focal plane, separately for odd and even bands, is averaged and plotted with respect to the integration times. Following this method, we obtain four different rates of DC with respect to which we study the response of the single pixels on the odd and even columns on each of the filters. A linear fit is applied, obtaining the offset and slope of the average DC curve with relative standard deviations. Thresholds are set at three times the computed standard deviations. This process is repeated for each pixel on the detector; the offset and slope of each fit are then compared to the established thresholds. A pixel of the focal plane is deemed defective if the difference between the parameters of its DC curve and the reference slope and/or offset exceeds the thresholds. The results of this analysis are reported in Table 3. The Delta stands for the difference between the slope of the single pixels’ DC trend vs. the average response of the FPA.

**Figure 11:**
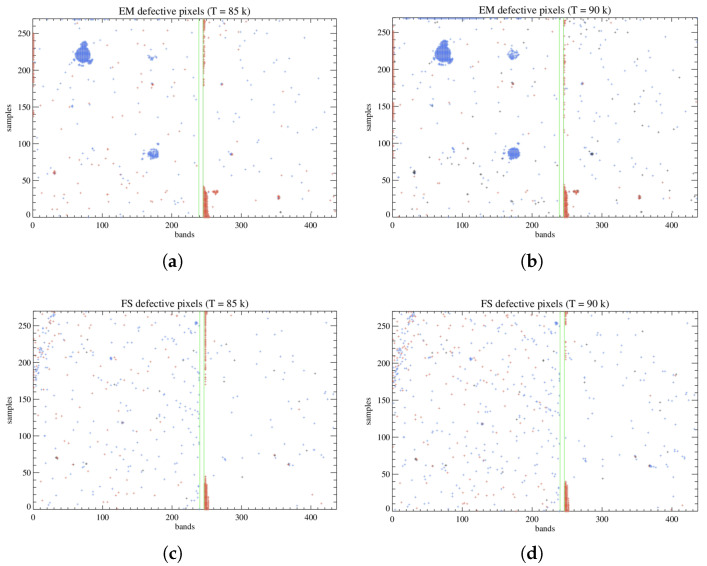
(**a**) Defects detected for the EM at T = 85 K. The defects exceeding the set thresholds negatively are represented in red, while the ones exceeding it positively are represented in blue. The hot pixels and dead pixels are shown in black. The green lines define the borders of the filter’s dead zone. (**b**) Same but for T = 90 K. (**c**,**d**) Same as (**a**,**b**) but for the FS detector.

For the EM model the amount of defective pixels detected ranges between 1251, for a detector temperature of 85 K, and 1599 for 90 K (accounting for 1.06–1.35 % of the total), while the FS shows 660 defects at 85 K and 761 at 90 K (0.56–0.64 %) (Figure 11).

As an additional analysis, we make a distinction between the pixels that exceed the preset slope threshold in positive and in negative, marked as “Delta” in Table 3. This latter group is thus characterized by a lower DC trend compared to the FPA average and therefore higher sensitivity and wider dynamic range with respect to the other set of faulty pixels [10]. Most defects detected on the FS belong to the lower-DC group, while the EM shows a clear majority of the higher-DC type. We also differentiate a third variety of defective pixels, detected in very small numbers and almost exclusively in the B filter region, which are stuck at a fixed value across the entire range of integration times and are identified due to their offset exceeding the threshold. Most of these are “hot pixels” as they remain fixed at a signal of 16,380–16,382 DN, corresponding to saturation. A very small percentage are instead identified as “dead pixels” as they exhibit no signal at all, resting at a value of 0 DN.

### 3.4. Detector Response Linearity

**Figure 12:**
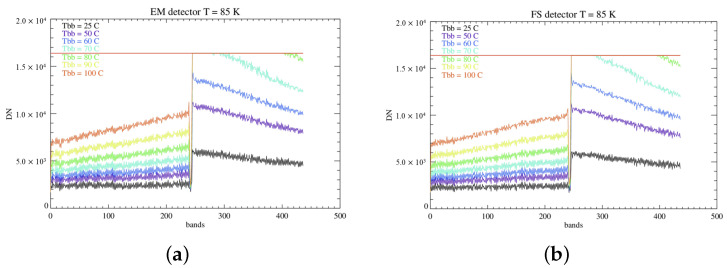
(**a**) Signal mediated along the samples across the A and B filters on the EM detector at T = 85 K in the range of temperatures of the black body. The gap between the intensity of the signal measured on the two filters increases with the temperature, with the B filter beginning to saturate at 70 °C. (**b**) Same, but for the FS detector.

The extended black body signal is employed to test the linearity of the detectors’ radiometric response by providing a reference IR flux. The black body is positioned at a distance of 3 cm from the dewar aperture, and the filter wheel is placed in the open window configuration. Maintaining an integration time of 5 ms, the black body radiance ($BB(\lambda ,T_{BB})$) is first acquired with power turned off for background signal recording: in this case, its temperature matches the room temperature ($T_{BB}=$ 25 °C). After this step, the BB is turned on, and its temperature is progressively increased at 10 °C steps, starting at 50 °C up to 100 °C. It is observed that at 70 °C the response across the B filter begins to saturate (Figure 12). For this reason, we focus the study of the detector’s response across the A filter, which covers the 2–3.9 μm spectral region.

The response across the filter is averaged, and the contribution of the DC and thermal background is removed by subtracting the signal measured at $T_{BB}=$ 25 °C. Finally, the response is normalized with respect to the corrected measure at $T_{BB}=$ 50 °C:

(2) $$BB_{corr}\left(T_{BB}\right)=\frac{BB\left(T_{BB}\right)-BB\left(T_{BB}=25\;{}^{\circ}C\right)}{BB\left(T_{BB}=50\;{}^{\circ}C\right)-BB\left(T_{BB}=25\;{}^{\circ}C\right)}$$

The filter response is compared to the corresponding value as derived by employing the theoretical radiance ($W\;m^{-2}\;{\mathrm{srad}}^{-1}$ μm^−1^) emitted by the black body which is computed as described by Planck’s formula:

(3) $$BB_{t}\left(\lambda ,T\right)=\frac{2hc^{2}}{\lambda^{5}}\frac{1}{e^{hc/\lambda k_{B}T}-1}$$

where $h=6.62\times 10^{-34}$ J·s is Planck’s constant, $c\approx 2.998\times 10^{8}$ m/s is the speed of light in a vacuum, λ is the wavelength in μm, $k_{B}=1.38\times 10^{-23}$ J/K is Boltzmann’s constant and *T* is the blackbody temperature in Kelvin.

**Figure 13:**
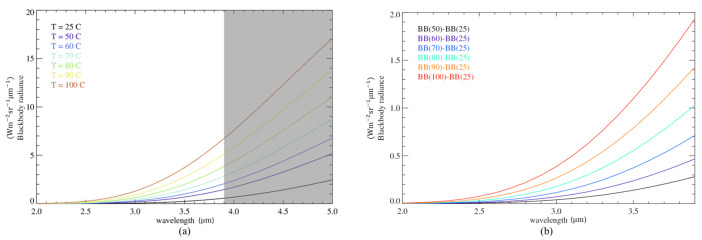
(**a**) Radiance emitted by the black body across the 2–5 μm spectral range and in the interval of temperatures considered. The grey region signals the wavelengths outside the range covered by the filter. (**b**) Radiance curves coming from the correction of the signals at black body temperatures of 50–100 °C with respect to the 25 °C radiance.

**Figure 14:**
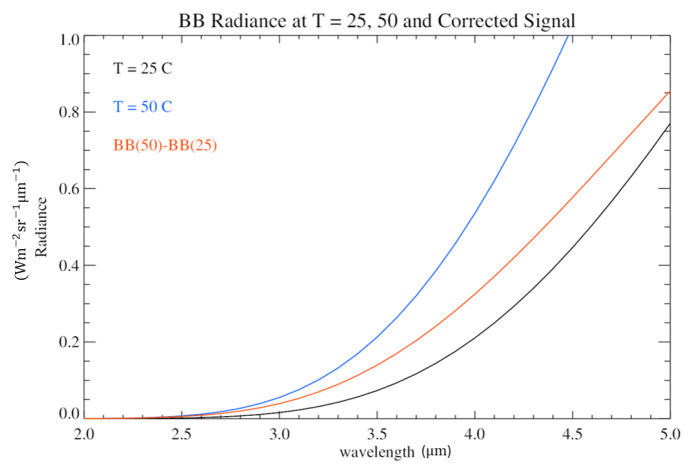
Black curve: theoretical black body radiance at T = 25 °C. Blue curve: theoretical black body radiance at T = 50 °C. The red curve shows the signal resulting from the difference between the two radiances. This latter signal is then integrated in the 2–3.9 spectral range, and the obtained value is compared to the corresponding averaged and corrected signal measured by the detector.

Considering this relation, we calculate the radiation emitted by the black body in the 2–5 μm spectral range (Figure 13). Following the same correction and normalization procedure applied for the detector measurements, the theoretical radiance is subtracted from the signal at 25 °C (an example of the signal resulting from this process is represented in Figure 14). The curves obtained from this operation are successively integrated in the 2–3.9 μm spectral interval and are finally normalized with respect to the integral of the curve $BB_{t}\left(T_{BB}=50\;{}^{\circ}C\right)-BB_{t}\left(T_{BB}=25\;{}^{\circ}C\right)$:

(4) $$BB_{t,corr}\left(T_{BB}\right)=\frac{\int_{2\mu m}^{3.9\mu m}\left(BB_{t}\left(T_{BB}\right)-BB_{t}\left(T_{BB}=25\;{}^{\circ}C\right)\right)}{\int_{2\mu m}^{3.9\mu m}\left(BB_{t}\left(T_{BB}=50\;{}^{\circ}C\right)-BB_{t}\left(T_{BB}=25\;{}^{\circ}C\right)\right)}$$

**Figure 15:**
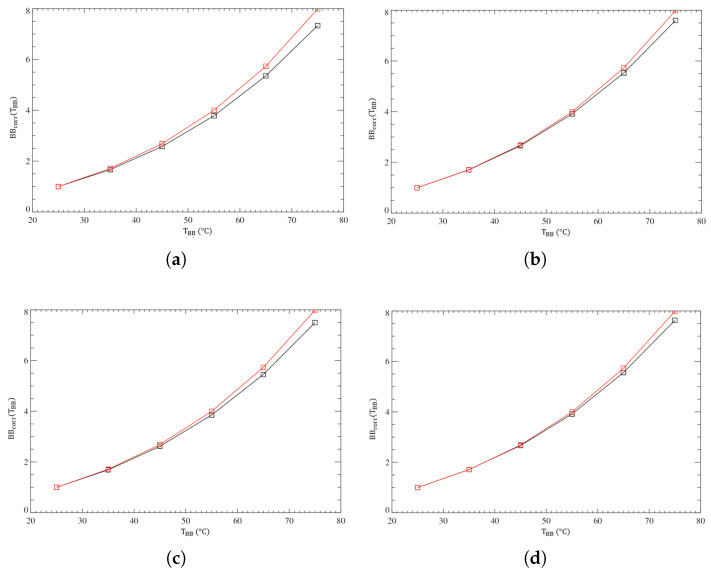
(**a**) Black body integrated radiance (red curve) vs. $T_{BB}$ compared to the EM normalized response (black curve) for a detector temperature of 85 K. (**b**) Same but for a detector temperature of 90 K. (**c**,**d**) Same as (**a**,**b**) but for the FS.

The theoretical radiance as a function of $T_{BB}$ obtained following this procedure is compared to the adjusted response of the detectors (Figure 15).

As shown in the plots, the A filter response proves a good compatibility with the black body radiance in both detectors and for both temperatures of 85 K and 90 K, only starting to diverge from the expected trend for higher temperatures.

## 4. Discussion

### 4.1. Dark Current Analysis

The dark current (DC) signal is a noise intrinsic to the material of the detector, generated by the thermal excitation of carriers in the semiconductor, which is measured in the absence of incoming radiation. Due to the presence of additional energy states introduced by impurities and related to lower activation energies, dark current is usually more intense in extrinsic sensors, like JIRAM’s photodetector models, compared to intrinsic systems [11]. The noise generated by the DC can significantly affect a sensor’s performance in signal detection, especially for instruments observing in the infrared, where cryogenic operating temperatures are employed in an attempt to mitigate it. An accurate measurement of the DC signal is consequently very important because it has a strong dependence on the detector’s temperature and the exposure time [12].

The causes of the features observed on the EM DC frames (Figure 4a,b) are traceable to the detector’s production process: the arc structures are probably attributable to the substrate removal procedure applied to improve the detector’s quantum efficiency, and the circular patterns are local defects probably introduced during the gluing process of the photosensitive element on top of the ROIC.

The decrease of signal which is visible across an approximately 30-pixel-wide strip and interests the whole length of the detector (along the spectral dispersion axis) on both FS-EM data (Figure 4a–d) can instead be explained as an intrinsic characteristic of the laboratory setup: the area shows a significantly lower DC signal and appears identical on the two models of photodetector. This seems to denote the presence of a slightly colder region on the focal planes, which we have attributed to a partially defective thermal connection between the sensors and the cold plate. Therefore, it is entirely related to the laboratory components encasing the FPAs during these specific measurements, rather than a product of any intrinsic spatial variability of the detectors’ performance. Since any future test on the sensors will be carried out with the aid of a new set of instruments and a different laboratory configuration, we considered this observed feature negligible for the purposes of our study.

**Figure 16:**
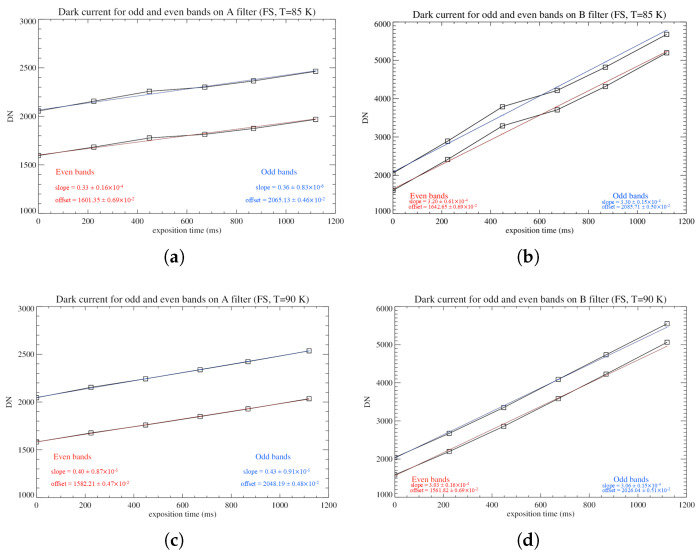
(**a**) Average dark current (DN) recorded on odd and even bands of pixels across the A filter, on the FS detector at T = 85 K, for exposure times from 0 ms to 1120 ms. The red curve is the linear fit applied to the signal measured on even bands; the blue curve is the linear fit applied to the signal measured on the odd bands. (**b**) Average DC and linear fits of the odd and even bands across the B filter. (**c**,**d**) Same as (**a**,**b**) but for T = 90 K.

Before proceeding with the evaluation of the dark current noise affecting the detectors, we check for the presence of the odd-even effect (as described in Section 3.1) to verify the necessity of splitting the pixels of the focal plane into distinct populations while performing further analyses. A further study is performed—here are reported the results for the FS detector—comparing the average DC measured on the odd and the even bands for different exposure times, separately for the A and B filters (Figure 16).

The DC signal follows, in all cases, a linear, smooth trend to which a linear fit is applied. The DC shows a very similar slope for the odd and even bands (with an average slope of 0.3–0.4 across the A filter and 3.0–3.3 on the B filter), so that the relative offset between the two trends stays sufficiently constant with respect to the exposure time. The offset between the DC measured on each filter for each integration time can be calculated and averaged, and then subtracted to correct the odd-even effect. The average relative offset, in DN, between the DC rates recorded on the odd and even bands of the focal plane, with associated uncertainty, is reported in Table 4. The values have been obtained by averaging the difference between the signal recorded on the odd and even bands on each filter and for each integration time. The uncertainty has been calculated through the error propagation of the standard deviation errors for each population.

### 4.2. Defective Pixels Analysis

Comparing the results of our analysis (Table 3) to the 2009 Raytheon tests, which measured 726 defects on the EM detector and 811 on the FS, we can infer that the years of inactivity have slightly degraded the detectors. The discrepancy in the number of faulty pixels can also be attributed to the different methods of detection employed: during the Raytheon tests, the defects were tracked, taking into consideration the quantum efficiency, ROIC noise, and well capacity, besides the DC measurements. Additionally, in the EM case, the 2009 analysis seems to have been carried out before the detector integration was complete, as the focal plane does not show the high-DC circular patterns we detected, and the related defective pixels are not counted.

Despite the slight deterioration of the focal planes, the percentages of faulty pixels are acceptable for the accomplishment of MIST-A’s objectives. It should also be taken into consideration that while this statistic covers the entire 438 × 270 pixels area of the detectors, the instrument’s operations will actually involve a smaller 336 × 256 pixels-wide window, so the effective number of defects affecting MIST-A’s performance will be lower.

## 5. Conclusions

The tests have confirmed that both detectors are still operational after a 15-year period of inactivity. When comparing the results of our analysis to the tests performed in 2009–2010, both models exhibit a certain degree of degradation, noteworthy in the EM detector, but still maintain sufficiently good performances and mostly preserve their original properties. As JIRAM’s scientific requirements are akin to MIST-A’s—both spectrometers observing in the infrared with a 2–5 μm operating spectral range—and these detectors proved to be able to fulfill the former’s objectives, we consider them suitable for MIST-A’s goals. Due to the absence of arc and circular structures, the lower slope of the DC curve, and the inferior number of defective pixels detected, the FS detector is considered to have a better quality with respect to the EM detector and appears to be more suited to be integrated on MIST-A. These results lead us to choose the FS as MIST-A’s Flight Model, while the EM is going to be kept as the instrument’s Flight Spare.

The tests that have been reported here will be repeated for the FS detector once it is installed on the flight model of the MIST-A instrument with its appropriate electronics unit. The detector will also be further analyzed in order to gain a more complete overview of its performance. The calibration campaign of MIST-A is going to be performed by the end of 2026 at the Institute for Space Astrophysics and Planetology (INAF-IAPS) facilities in Rome; currently, the laboratory setup is being integrated and tested. Once the calibration is completed, the flight hardware and Electrical Ground Support Equipment (EGSE) of the instrument are going to be delivered to the Laboratory for Atmospheric and Space Physics (LASP) for spacecraft integration. After the characterization tests at IAPS, MIST-A’s detector is not going to be further analyzed until the Early Operations mission phase after launch, during which the aliveness checkouts and preliminary performance characterizations of the MBR Explorer’s payload are taking place. 

## Figures and Tables

**Table 1:** Most frequent DC values recorded on the odd and even bands.

		EM	FS
T = 85 K	Even bands:	1616 DN	1600 DN
	Odd bands:	2116 DN	2050 DN
T = 90 K	Even bands:	1616 DN	1550 DN
	Odd bands:	2166 DN	2000 DN

**Table 2:** Offset (q) and slope (m) of the linear fits of the DC.

		EM	FS
T = 85 K	q (DN)	1904.29 ± 0.81	1851.11 ± 0.45
	m (DN/ms)	1.67 ± 0.14 $\times \;10^{-2}$	1.62 ± 0.10 $\times \;10^{-2}$
T = 90 K	q (DN)	1925.39 ± 0.83	1803.43 ± 0.46
	m (DN/ms)	2.26 ± 0.16 $\times \;10^{-2}$	1.59 ± 0.10 $\times \;10^{-2}$

**Table 3:** Defective pixels on the EM and FS detectors at T = 85, 90 K.

	EM (85 K)	EM (90 K)	FS (85 K)	FS (90 K)
Total Number of Defective Pixels	1251	1599	660	761
Positive Delta	743	1070	285	390
Negative Delta	505	436	348	339
Fixed Value	3	93	27	32

**Table 4:** Average relative offset between the DC rates on the odd and even bands.

	A Filter	B Filter
T = 85 K	485.33 ± 0.96	487.46 ± 1.61
T = 90 K	480.49 ± 0.87	486.67 ± 1.63

## Data Availability

The data that support the findings of this study are available from the corresponding authors upon reasonable request.
